# Carabid beetle (Coleoptera, Carabidae) richness, diversity, and community structure in the understory of temporarily flooded and non-flooded Amazonian forests of Ecuador

**DOI:** 10.3897/zookeys.1044.62340

**Published:** 2021-06-16

**Authors:** Kathryn N. Riley Peterson, Robert A. Browne, Terry L. Erwin

**Affiliations:** 1 Department of Biology, Wake Forest University, Winston-Salem, NC, USA Wake Forest University Winston-Salem United States of America; 2 Department of Natural Sciences, Pfeiffer University, Misenheimer, NC, USA Pfeiffer University Misenheimer United States of America; 3 Department of Entomology, National Museum of Natural History, Smithsonian Institution, Washington, DC, USA Smithsonian Institution Washington United States of America

**Keywords:** Amazon, flight intercept traps (FITs), forest type, ground beetles, hand sampling, Yasuní rainforest

## Abstract

Although tropical regions harbor the greatest arthropod diversity on Earth, the majority of species are taxonomically and scientifically unknown. Furthermore, how they are organized into functional communities and distributed among habitats is mostly unstudied. Here we examine species richness, diversity, and community composition of carabid beetles (Coleoptera: Carabidae) and compare them between flooded (FP) and non-flooded terra firme (TF) forests in the Yasuní area of Ecuador. The forest understory was sampled using flight intercept traps (FITs) and systematic hand collections at night in June and July 2011 and 2012, and FITs in October and November 2011. A total of 1,255 Carabidae representing 20 tribes, 54 genera, and 143 morphospecies was collected. Mean number of individuals and mean species richness did not differ significantly between FP and TF; however, numbers of Cicindelini (tiger beetles) and Pentagonicini were higher in TF forest while numbers of Lachnophorini and Scaritini were higher in FP forest. Overall, FP had significantly higher rarefied richness but extrapolation of rarefaction curves using the Chao1 nonparametric diversity estimator show that this difference may decrease with additional sampling. The inverse Simpson index was significantly higher for FP than TF forest. Nonmetric multidimensional scaling (NMDS) ordination and dissimilarity coefficient values show that FP and TF forests maintain unique assemblages with minimal overlap in community composition. Given ongoing anthropogenic pressures, particularly petroleum extraction, and those resulting from climate change, a greater understanding of the richness, diversity and community assemblages of Yasuní rainforest are needed to better conserve the fauna of this megadiverse area of Amazonia.

## Introduction

Insects and their arthropod relatives dominate eukaryotic global biodiversity (Erwin 1982; Hammond 1992; Samways 1993; Basset et al. 2012). Although approximately 1.30 million arthropod species have been described, >80% of which are insects (Zhang 2013), estimates by Hamilton et al. (2013) and Stork et al. (2015) suggest that 60–80% of the 6.1 million predicted arthropod species await description (Erwin and Johnson 2000; Dunn 2005; May 2010; Hamilton et al. 2010, 2013; Stork 2018). Tropical habitats harbor the greatest arthropod biodiversity and considerably more empirical work is needed just to describe currently unnamed tropical arthropods (May 2010; Mora et al. 2011; Hamilton et al. 2013; Stork 2018). Arthropods play vital roles in ecological processes and are major components of highly diverse ecosystems (Kim 1993; Samways 1993). With current rates of deforestation, forest degradation, and predicted changes associated with climate change such as aridization, many tropical forest species will disappear before they are described (Kellert 1993; Kim 1993; Samways 1993; Godfray et al. 1999; Dunn 2005; Finer et al. 2008; Kim et al. 2015; Kirichenko-Babko et al. 2020; Wagner 2020).

Greater comprehension of tropical arthropod diversity and communities is needed for realistic global diversity estimates, to discern ecological patterns including species distributions and to support conservation strategies (Erwin 1991a; Oliver and Beattie 1996; Carlton et al. 2004; Rohr et al. 2007; Erwin and Geraci 2009; May 2010; Mora et al. 2011; Stork 2018). We have only a limited understanding of the structure, general patterns, and assembly rules for arthropod communities in tropical forests, even across broad scale environmental gradients. Nonetheless, species turnover across habitats is a central aspect of Amazonian diversity (Pitman et al. 2001; Condit et al. 2002; Draper et al. 2019) and arthropod assemblages are expected to vary markedly across lowland tropical forests (Erwin 1983a; Lucky et al. 2002; Dyer et al. 2007). Fine et al. (2013), for example, found high turnover of herbivorous insects using a single tree species across two Amazonian forest types, as well as high turnover among tree species within the same habitat. Additional research is needed for non-herbivore arthropods to improve estimates of overall turnover in tropical forests (Kitching 2006; Fine et al. 2013).

Amazonian forests have been classified into broad types based on forest structure, drainage, floristic composition, geology, and soils (Prance 1979; Duivenvoorden and Lips 1995; Herrera-MacBryde and Neill 1997; Baraloto et al. 2011; Myster 2016). Two major forest types in Amazonia are floodplain forests (FP) and upland non-flooded terra firme (TF) forests (Pitman 2000; Myster 2014, 2016). Terra firme forests are the most widespread forest type in the Amazon Basin while FP forests are riparian forests subject to episodic inundations by adjacent rivers during periods of high precipitation or upland Andean precipitation and/or snowmelt (Prance 1979; Junk et al. 1989; Worbes 1997; ter Steege et al. 2000; Parolin et al. 2004; Junk and Piedade 2010; Myster 2014, 2016). Previous studies show that both forest types are rich in species but TF forests are generally more species rich than FP forests for many taxa (Campbell et al. 1986; Balslev et al. 1987; Gascon et al. 2000; Haugaasen and Peres 2005; Myster 2016; Bredin et al. 2020) including arthropods: spiders (Höffer 1997), ants (Wilson 1987; Majer and Delabie 1994; Mertl et al. 2009), canopy arthropods (Erwin 1983a; Adis et al. 1984, 2010) and terrestrial arthropods in general (Adis 1997; Adis and Junk 2002; Lamarre et al. 2016). However, only a relatively small amount of comparative data is currently available in Amazonian forests, particularly for groups of understory Coleoptera (Adis and Junk 2002).

Few ecological studies have compared understory arthropod diversity and species assemblages from different forest types within Amazonia (Adis 1981; Pearson and Derr 1986; Majer and Delabie 1994; Zerm et al. 2001; Mertl et al. 2009; Lamarre et al. 2016; Santos-Silva et al. 2018). Even fewer report forest understory diversity and community data for the highly diverse Carabidae (Irmler 1979a, b; Zerm et al. 2001; Lamarre et al. 2016; Cajaiba et al. 2018), which includes a range of trophic roles including predaceous, predominately phytophagous and omnivorous species as well as some with specialized modes of nutrition (Thiele 1977; Lövei and Sunderland 1996). Current knowledge of Amazonian carabids in the understory of FP forests is largely based on research within Central Amazonia where flooded forests are annually inundated by several meters of water for several months a year (Irmler 1979a, b; Adis 1981; Paarmann et al. 1982; Zerm et al. 2001). In contrast, our study focuses on a forested area within the highly diverse western Amazon which experiences less severe inundation events, both in duration and frequency, than in Central Amazonia (Burnham et al. 2001; Hoorn et al. 2010). For ground-level understory Carabidae of temporarily flooded (FP) and non-flooded TF forests in the Ecuadorian Amazon, we investigated the following: differences in species abundance and richness, patterns in the abundance and distributions of common and rare species, and characterization of carabid assemblages including identification of taxa characteristic to each forest type.

In contrast to findings reported for other taxa, we predicted that carabids of FP forests will have greater species richness and diversity than adjacent TF forests. This prediction, in part, stems from the taxon pulse concept (Erwin 1979, 1985) which suggests that waterside habitats were a common habitat for primitive carabid lineages. Therefore, due to historical habitat associations, riparian FP forests habitats may harbor higher numbers of species and diversity of carabid beetles compared to TF forests. Higher richness of Carabidae was reported in flooded forests compared to TF forests in Erwin and Adis (1982), Pearson (1984), and Pearson and Derr (1986). Additionally, higher species richness has been reported for forest-dwelling carabid beetles in response to disturbances (e.g., clear-cutting) (Erwin 1979; Niemelä et al. 1993; Koivula et al. 2002; Magura et al. 2006; Riley and Browne 2011). Furthermore, disturbance associated with flooding events may also foster increased carabid species richness through mechanisms such as a reduction in competition (Connell 1978; Erwin and Adis 1982; Zerm and Adis 2001a, b). It is expected that carabid species composition will differ between the forest types due to differences in plant composition, temporary flooding events, forest structure and resource availability (Campbell et al. 1986; Balslev et al. 1987; Worbes 1997; Bredin et al. 2020).

## Materials and methods

### Description of the study area

**Figure 1.**
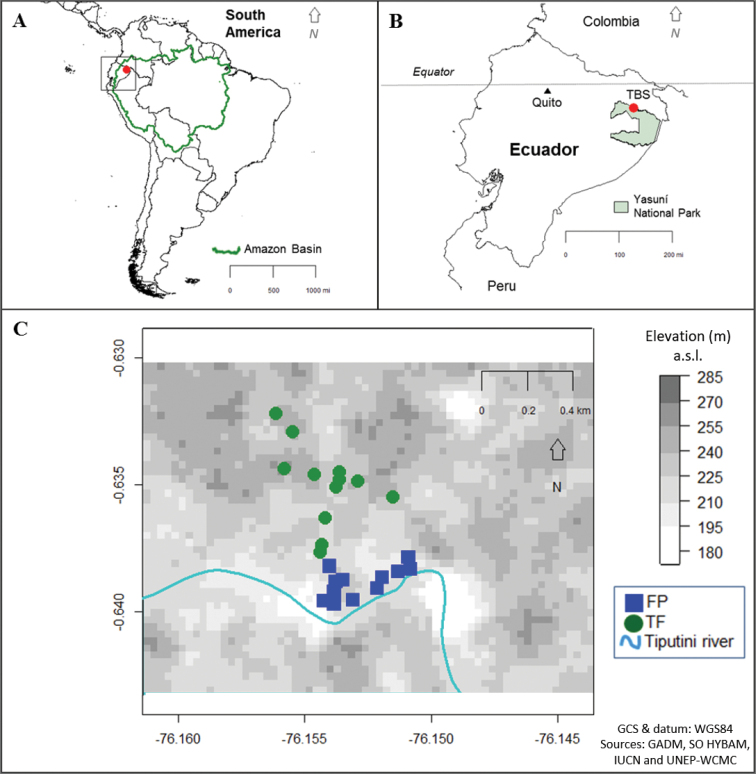
Maps of the field sites **A** country boundaries of South America with the Amazon Basin indicated by the heavier outline **B** Ecuador with the locations of TBS as a red circle and the boundaries of Yasuní National Park shaded **C** a DEM of the study area. The 24 sampling sites are indicated, with blue squares representing FP forest sites and green circles for TF forest. Latitude and longitude (in DD) shown along x and y axes. Maps generated through R packages: ‘raster’ (Hijmans 2015), ‘sp’ (Pebesma and Bivand 2005; Bivand et al. 2013), ‘GISTools’ (Brunsdon and Chen 2014), and ‘maps’ (Becker et al. 2015).

This research was conducted at Tiputini Biodiversity Station (TBS) (0°37'55"S, 76°08'39"W; 190–270 m elevation a.s.l.) (Fig. 1A, B) located in the Ecuadorian Amazon, within the province of Orellana. TBS consists of 650 ha of moist tropical rainforest and borders the megadiverse Yasuní National Park (Bass et al. 2010; Myster 2016). The forest surrounding TBS has experienced relatively minimal anthropogenic disturbance compared to other parts of the Amazon. The area has average annual temperatures of 24–27 °C and high yearly rainfall of ~ 3200 mm. The climate is considered aseasonal compared to other areas in Amazonia, as no month receives < 100 mm of rain. However, there are seasonal patterns in precipitation with rainier months (‘wetter season’) from April through June and September–October and drier periods (‘drier season’) in August and November–January (Pitman 2000; Blake et al. 2011; Myster 2016).

The area is relatively flat but several ridges, ~ 25–50 m higher than watercourses, add slight topographic variation. Approximately 90% of the landscape surrounding TBS is non-flooded TF which occurs along the slopes and tops of ridges. Rivers dissect these large blocks of TF forest creating narrow bands of FP forest which comprise ~ 2% of the study area (Pitman 2000; Pitman et al. 2001; Guevara et al. 2012; Pitman et al. 2014; Myster 2016). The palm, *Iriartea deltoidea* Ruiz & Pavn is a dominant tree species in both FP and TF forests near TBS (Pitman et al. 2001; Myster 2014), which are described in more detail by Burnham et al. (2001), Pitman et al. (2001), and Myster (2014, 2016).

Floodplain forest occurs within a relatively narrow area along the Tiputini River, which has been described as a mixed-water river (contributions of white and black water), or other times as white water (várzea), with a relatively high sediment load (Pitman 2000; Myster 2016). The height and volume of the Tiputini River is most strongly influenced by rainfall and snowmelt in the Andes Mountains (Prance 1979; Pitman 2000; Burnham 2002). See Appendix A for information about mean annual river heights for the years relevant to this study. Forests are inundated by the river for various lengths of time depending on elevation and distance from the river (Burnham et al. 2001; Pitman et al. 2001; Mertl et al. 2009). Previous studies have estimated that the floodplains around TBS are inundated (< 3 m) 1–3 times a year with inundations usually lasting less than a week (Pitman 2000; Burnham et al. 2001; Leimbeck and Balslev 2001; Burnham 2002; Mertl et al. 2009; Myster 2016).

Tiputini Biodiversity Station is located on a geologically young landform consisting of fluvial deposits (red clays and alluvium) originating from the Andes Mountains and are rich in exchangeable bases (Hoorn et al. 2010). Soils of TF have been classified as Ultisols which are clayey, acidic, and high in iron and aluminum (Pitman 2000; Sombroek 2000; Tuomisto et al. 2003). FP soils are less acidic, lower in iron and aluminum but higher in other nutrients (Ca, Mg, K, Na, P) and organic carbon compared to TF soils (Kapos et al. 1990; Duivenvoorden and Lips 1995; Pitman 2000).

### Collection of Carabidae

Carabid beetles were sampled during the wetter months of June and July in 2011 and 2012 and during the transition to the drier season in October and November of 2011. Two sampling techniques, flight intercept traps (FITs) and hand sampling, were deployed sites in the forest understory, 12 in each of the two forest types (Fig. 1C). Both methods were employed in the rainier seasons but for logistical reasons only FITs were employed in the transitional season in 2011. The number of FIT trapping days varied slightly between forest types due to flooding events in the 2011 wet season, ranging from 33–49 days in the 2011 wetter season, 30–31 days in the 2012 wetter season and 29 days in the 2011 transitional season.

Each FIT consisted of PVC tubes, screen netting, a plastic awning (to prevent rain and debris from entering the trays) and plastic collection trays. Screening was stretched between two 1 meter PVC tube, with a 1 m support PVC tube across the top of the trap to create an ~ 1 m^2^ flight barrier. See Riley et al. (2016) for additional details on FIT materials and implementation. Plastic trays were placed underneath the flight barrier to collect specimens and filled with water and biodegradable soap. Sampling trays were emptied every other day by filtering the contents through a metal strainer with mesh openings < 1 mm.

Since the majority of Carabidae are active at night, hand sampling occurred between 8:30 pm and 10:30 pm with the use of headlamps. This included active searching of the ground (leaf litter, etc.), tree trunks and vegetation (up to eye level), and decaying logs. A total of seven hand sampling events was performed at each of the 24 sampling sites durng two field seasons, four at each site in 2011 and three in 2012. Each sampling event consisted of 0.5 hour of search effort, mostly along a trail, within 100 meters of the FIT for each site. The first author participated in every hand sampling event with the assistance of one or two trained volunteers. Immediately after capture, carabids were preserved in 95% ethanol.

The main goal of this study was to compare richness, diversity, and assemblages of Carabidae between FP and TF forest types; therefore, data from both FITs and hand collecting were simply pooled by each sampling site for the analysis presented in this paper. However, we recognize that collection techniques operate differently and strongly influence the composition of fauna sampled (Gadagkar et al. 1990; Kitching et al. 2001; Missa et al. 2009). In a separate paper, Riley et al. (2016), we compared numbers of individuals, species, diversity, and community-level analyses between hand sampling and FITs for the two forest types, as well as reported taxa-specific results.

### Identification of carabid beetles

Carabid beetles were sorted using a stereo microscope and identified to genera and morphospecies. Since a species-level taxonomic key is not available for the study region, a genus-level key for French Guyana Carabidae (Erwin et al. 2012) was used, with subsequent identification to morphospecies and taxonomic confirmation by TE^†^. Only two species, a widespread Neotropical tiger beetle *Odontocheila cayenensis* Lam. and the hiletine, *Eucamaragnathus batesi* Chaudoir, could be confidently referred to described species. In addition, the single oodine specimen collected could be confidently referred to *Oodinus* but could not be confidently determined as belonging to either *O. piceous* Motschulsky, *O. amazonas* Chaudoir, or *O. limbellus* Chaudoir. Otherwise, identifications were restricted to morphospecies. The use of morphospecies for Neotropical arthropods has been successful in ecological studies and is the most feasible option for an extraordinarily speciose region, with the majority of species not described by science (Oliver and Beattie 1996; Stork et al. 2008; Grimbacher and Stork 2009). Since the vast majority of the data set consists of morphospecies, we will use the term morphospecies unless specifically referencing one of the described species mentioned above. Voucher specimens representing taxa collected in this study will be deposited at Museo de Historia Natural, La Escuela Polytecnica Nacional (**EPNC**) in Quito, Ecuador. Abbreviations used in this paper are as follows:

**ABL** apparent body length;

**ACE** abundance-based coverage estimator, ACE;

**DEM** digital elevation map;

**FITs** flight intercept traps;

**FP** floodplain;

**1/D** inverse Simpson index, a Hill diversity number;

**IndVal** an indicator statistic, developed by Dufrene and Legendre (1997);

**ISA** Indicator Species Analysis;

**N** number of individuals;

**NMDS** nonmetric multidimensional scaling;

**Pentb** the most commonly collected cicindelid *Pentacomia* morphospecies;

**S** number of morphospecies (i.e., species richness);

**S.E.** calculated standard error;

**E_1/D_** Simpson’s evenness;

**TBS** Tiputini Biodiversity Station;

**TF** terra firme.

### Statistical analyses

As a precursor to our statistical comparisons of FP and TF forests, the data set was tested for spatial autocorrelation. First, a multivariate Mantel correlogram was constructed (Mantel 1967; Oden and Sokal 1986; Sokal 1986; Borcard and Legendre 2012; Legendre and Legendre 2012) using the ‘vegan’ package in R (Oksanen et al. 2016; R Core Team 2016). Inputs for this analysis were the Bray-Curtis dissimilarity for species assemblages and the Cartesian coordinates of the sampling sites as determined using R package ‘SoDA’ (Chambers 2013). If a linear trend was detected, species data were Hellinger-transformed and detrended prior analysis to satisfy normality and the second-order stationarity condition (Borcard et al. 2011). Sturge’s rule was used to determine the number of distance classes (Borcard et al. 2011). Following 5,000 permutations the significance of the normalized Mantel statistic for the distance classes were evaluated using Holm correction for multiple tests (Holm 1979; Borcard et al. 2011; Goslee and Urban 2013). Autocorrelation analyses were completed using R package ‘vegan’ (Oksanen et al. 2016; R Core Team 2016). To correct for a linear trend (F = 3.08, df = 2, P < 0.01), data were Hellinger-transformed and detrended prior to construction of the Mantel correlogram. There were six distance classes, with the smallest ranging from 0 to 32.5 meters; however, no significant spatial correlations were detected so all sample sites were treated as independent sites in all statistical analyses.

Data from both sampling techniques were simply pooled by sampling site to determine the N, S and 1/D (Simpson 1949; Jost 2006; Chao et al. 2014). To detect changes at higher taxonomic levels, the numbers of individuals and species for each carabid tribe were also analyzed. For the analyses listed above, values for FP and TF sites were compared using a two-sample t-test with equal variance unless assumptions of the test were not met. If the assumption of homogeneity was violated, we used two-sample t-tests with a Welch correction. When data failed to meet the assumption of normality, a Wilcoxon rank sum test was used.

Mean body length was compared between forest types using ABL. This is the length from the extreme anterior point of the mandible to apex of elytra and is a standard measurement used in many ecological studies to provide a reliable estimate of overall size for Carabidae (e.g., Erwin and Kavanaugh 1981). For each ground beetle morphospecies, all intact specimens (i.e., the head, thorax, and abdomen were undamaged) were measured and, due to their greater abundance in the sample, at least 21% of the specimens for each tiger beetle (Cicindelini) morphospecies were measured. For each sampling site, mean ABL was determined by averaging the measurements of all individuals collected over the entire study period. The mean site ABL values for FP and TF sites were compared using a two-sample t-test with equal variance.

To account for differences in numbers of individuals collected between forest types and under-sampling bias, sample size was standardized by rarefaction which determines the expected number of morphospecies from a random subsample of individuals from the overall data set (Gotelli and Colwell 2001, 2011). Rarefaction curves for each forest type were constructed in ‘iNEXT’ using R software (Hsieh et al. 2015), using 500 randomizations to determine 95% unconditional confidence intervals (Chao et al. 2014). The x-axis was scaled to individuals to show the increases in number of morphospecies as more individuals were collected (Gotelli and Colwell 2001, 2011; Colwell et al. 2004, 2012). To estimate the number of undetected morphospecies, rarefaction curves were extrapolated by a factor of two from the smallest sample size between forest types (Colwell et al. 2012). The abundance-based non-parametric species richness estimator, Chao1 was used to extrapolate the rarefaction curves. Chao1 has been recommended as the lower bound of asymptotic richness (Chao 1984; Hortal et al. 2006; Colwell et al. 2012).

We attempted to account for estimator bias, by calculating two additional non-parametric diversity estimators with standard errors (randomizations = 1000) using the R package ‘vegan’ (Oksanen et al. 2016): the first order jackknife, Jack1 (Burnham and Overton 1979; Heltshe and Forrester 1983) and the abundance-based coverage estimator, ACE (Chao and Lee 1992; Chao and Yang 1993; Lee and Chao 1994). Estimators were selected based on high performance reported in previous studies (Chazdon et al. 1998; Brose and Martinez 2004; Walther and Moore 2005; Hortal et al. 2006; Gotelli and Colwell 2011), as well as the species abundance distribution and evenness of the data, all of which affect estimator biases. Since ACE accounts for the frequency of rare species, it is more appropriate for datasets with a high proportion of rare species as is common for studies examining carabid assemblages (Chao and Lee 1992; Chazdon et al. 1998; Belaoussoff et al. 2003). Jack1 has performed well in a variety of studies (Colwell and Coddington 1994; Walther and Moore 2005; Hortal et al. 2006; Basualdo 2011) with low bias for data with higher evenness and more mobile organisms (Palmer and Dixon 1990; Brose and Martinez 2004). Results from the three estimators were averaged to provide conservative values relating to sample completeness.

Simpson’s evenness (E_1/D_) values (Williams 1964; Smith and Wilson 1996; Payne et al. 2005) were calculated for each sampling site and the significance of the difference was determined by a two-sample t-test with equal variance. Log of relative abundance was plotted against morphospecies rank to compare morphospecies abundance distributions between FP and TF forests. In order to examine morphospecies commonness in the sample, morphospecies were assigned to three rarity categories based on the proportion of their abundance to the overall catch (relative abundance) over the entire sampling period with both forest types combined. Morphospecies were classified as ‘dominant’ if their relative abundance contributed > 10.0% of the total number of individuals collected. ‘Common’ morphospecies contributed 1.0–9.99% to the overall catch, and morphospecies were classified as ‘rare’ if they contributed < 1.0% to the total catch. Differences between FP and TF forests in numbers of individuals and morphospecies in these three rarity categories were analyzed through two-sample t-tests or, when t-test assumptions were not met, nonparametric Wilcoxon tests.

We used a generalized linear model with Poisson error distribution and a log link to test the hypothesis that common morphospecies should be collected from a higher number of sample sites. Significance was determined by analysis of deviance using the Wald chi-square test statistic. Linear modeling was completed using R packages ‘lme4’, ‘car’, and ‘multcomp’ (Hothorn et al. 2008; Fox and Weisberg 2010; Bates et al. 2015; R Core Team 2016). We determined whether the proportion of common and rare morphospecies were similar among tribes using a chi-square test. Dominant and common morphospecies were combined for this analysis (now termed ‘abundant’) to meet the assumptions of the test. Expected numbers for each category were determined by multiplying the total number of morphospecies collected in the tribe by the overall proportion of morphospecies in each rarity category (e.g., 125 of 143 morphospecies were rare, 87.4%).

Morphospecies assemblages for FP and TF were also compared using NMDS (Minchin 1987; Oksanen 2015). Bray-Curtis (abundance-based) and Jaccard (presence/absence) dissimilarity values were used as the distance measures. Non-metric multidimensional scaling (NMDS) is a robust unconstrained ordination method which makes no assumptions about the underlying nature of species distributions. Since the abundance mean-variance relationships were similar between FP and TF forests, untransformed abundance values of all morphospecies were used as the inputs for community-level analyses (Minchin 1987; Clarke 1993; McCune and Grace 2002; Nekola et al. 2008). To ensure solution stability, we used 100 runs for all NMDS analyses with random start points (Minchin 1987). Statistical significance between FP and TF distance matrices was determined using adonis, a robust permutational multivariate analysis of variance (permutations = 999) (Legendre and Anderson 1999; Anderson 2001; McArdle and Anderson 2001; Oksanen 2015). Dispersions within forest types were tested for multivariate homogeneity using function betadisper and TukeyHSD for the post-hoc test (Anderson 2006; Anderson et al. 2006; Oksanen 2015). All community-based analyses completed using R package ‘vegan’ and ‘MASS’ (Venables and Ripley 2002; Oksanen et al. 2016; R Core Team 2016) .

We sought to identify morphospecies that drove differences in community morphospecies assemblages using ISA. The IndVal statistic, developed by Dufrene and Legendre (1997), is insensitive to community-level beta diversity and does not consider absences as negative preferences (Dufrene and Legendre 1997; De Cáceres and Legendre 2009). Only morphospecies collected at least three sites (i.e., 25% of the number of sites in each forest type) over the entire sampling period were included in the ISA. Indicator value and statistical significance were determined using function multipatt in R package ‘indicspecies’ (permutations = 999). We used the Holm correction for multiple testing to reach experiment wide conclusions (Holm 1979; De Cáceres and Legendre 2009).

## Results

In total, 1,255 Carabidae were collected, representing 20 tribes, 54 genera, and 143 morphospecies (Table 1). Overall, FIT samples accounted for 64% (n = 805) of the total number of individuals collected and 55% (S = 79) of collected morphospecies were represented in FIT samples. Of the 438 individual tiger beetles collected, all but three individuals were collected by FITs (99%). Those collected by hand were resting on leaves next to the trail. In comparison, hand sampling at night accounted for 36% (n = 450) of the total number of individuals collected and 57% (S = 82) of collected morphospecies were represented in hand samples. Only 18 morphospecies were collected by both techniques, with 43% of the morphospecies collected exclusively by FITs and 45% exclusively by hand collecting. The goal of this paper was to compare the carabid fauna between FP and TF forest types, therefore samples from both collecting techniques were simply pooled by sampling site for the present analyses. Collections from hand samples and FITs in FP and TF forests are compared in detail by Riley et al. (2016) and the results, particularly the similarity in morphospecies accumulation curves for the two collection techniques in each forest type, suggest pooling these data will not distort the dataset or introduce any biases.

**Table 1.** Measures of numbers of individuals, morphospecies richness, and diversity of Carabidae collected for FP and TF forests.

Measure	FP	TF	Total
No. individuals	564	691	1255
Avg. individuals (± S.E.)	47.0 ± 5.0	57.6 ± 7.1	52.3 ± 4.4
No. morphospecies	96	79	143
Avg. morphospecies (± S.E.)	21.6 ± 2.0	16.9 ± 1.6	19.3 ± 1.3
No. tribes	20	14	20
No. genera	44	31	54
No. singletons	34	38	63
No. doubletons	15	8	22
			
*Richness estimators*			
Chao1 (± S.E.)	126.4 ± 13.7	159.1 ± 38.3	237.4 ± 32.9
Jack1 (± S.E.)	130.8 ± 12.9	122.1 ± 9.8	210.1 ± 17.8
ACE	131.4	147.0	248.6

**Figure 2.**
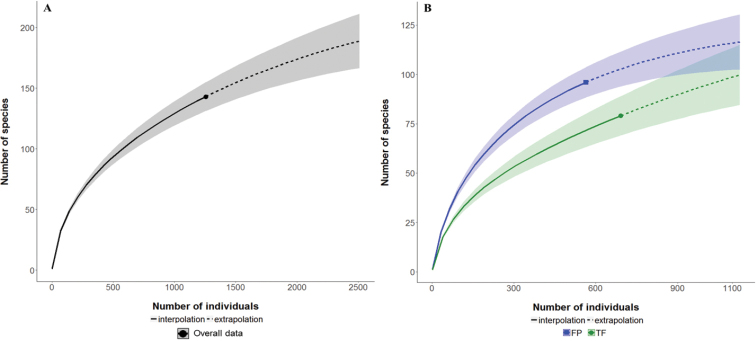
Carabid beetle rarefaction curves. Interpolation (solid lines) indicated by filled point markers represents the sampling extent of the current study. Extrapolation curves based on the Chao1 nonparametric diversity estimator are shown (dashed lines). Shaded areas depict unconditional 95% confidence intervals **A** the overall dataset with both forest types combined and richness extrapolated to n = 2,510 (twice the number of individuals collected) **B** rarefaction curves for FP forests (blue square) and TF forests (green circle) with sample size extrapolated to n = 1,128 (twice the number of individuals collected in FP forests). FP forests (96 ± 8.0) were significantly more species rich than TF forests (72 ± 8.1) at the rarefied sample size (n = 564). The extrapolated rarefaction curves suggest the difference in cumulative morphospecies richness between FP and TF will decrease as sample size increases.

**Figure 3.**
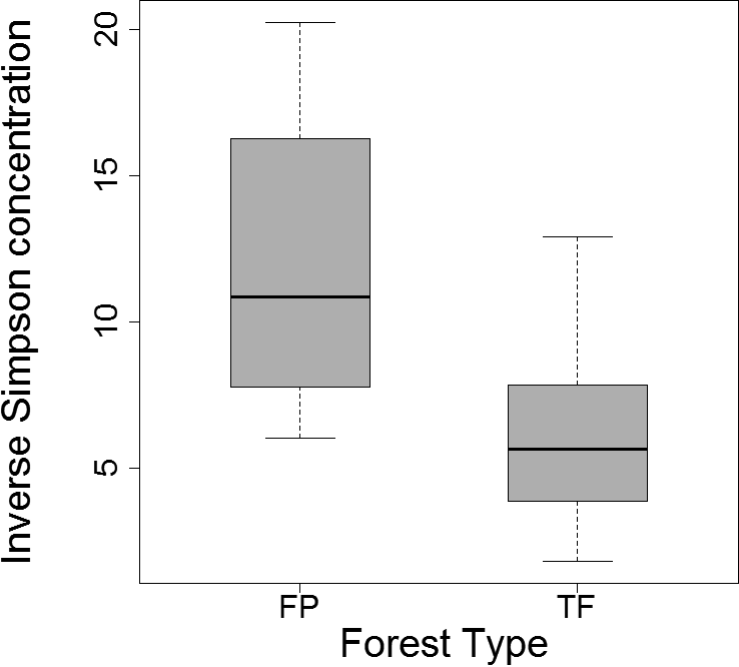
Inverse Simpson index (1/D) for FP forest and TF forest carabid beetle samples. FP forest had significantly higher 1/D values than TF forest (P = 0.003).

There were no significant differences between forest types in mean number of individuals or mean morphospecies number, although the difference in raw richness tended toward significance (P = 0.060; Table 1). The rarefaction curve for the pooled dataset did not approach an asymptote and the extrapolation from the curve through Chao1 estimates total morphospecies number as 189 ± 22.3 (mean ± S.E.) when sample effort was increased by a factor of two (Fig. 2A). Given the lack of overlap between the 95% unconditional confidence intervals for the rarefaction curves at n = 564, the rarefied richness in FP (96 ± 8.0) was significantly higher than in TF (72 ± 8.1) forests (Fig. 2B). However, the extrapolated rarefaction curves predict that the difference in cumulative richness between habitats will decrease as sample size increases. In fact, two of three nonparametric estimators predict that when fully sampled, true richness in TF will be higher than FP forest (Table 1). Average sample completeness across the three estimators were the following: FP (74.2%), TF (55.4%) and overall (62.0%). The inverse Simpson diversity index was significantly higher for FP than TF forest (t = 3.3, df = 22, P = 0.003; Fig. 3) emphasizing the greater importance of rare morphospecies in FP forest.

**Figure 4.**
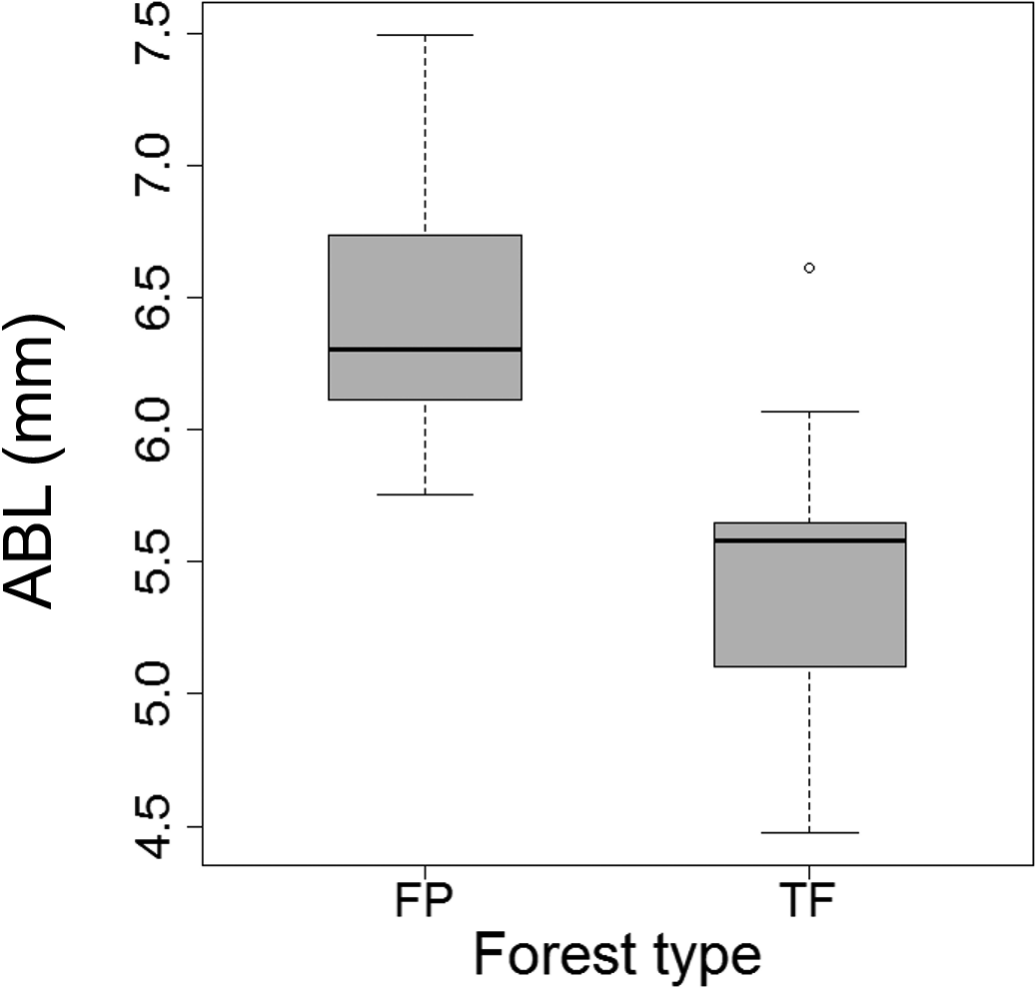
Body length (ABL) (mm) for carabid individuals collected in FP and TF forests, excluding Cicindelini. Body length for FP forest was significantly larger than TF forest (P < 0.001).

Carabidae body length ranged from 1.3 to 24.7 mm, with an overall mean (± S.E.) of 6.68 ± 0.19 mm for both forest types combined. There were no significant differences in mean body size between FP (6.78 ± 0.19) and TF individuals (6.59 ± 0.34). Tiger beetles have a relatively large body size (mean range: 9.9–16.1 mm) and because their abundance differed between habitats, ABL was also compared without Cicindelini. In this analysis, mean body length of non-cicindelid individuals collected at TF sites (5.47 ± 0.15 mm) were significantly smaller than individuals at FP sites (6.45 ± 0.16 mm) (t = 4.4, df = 22, P < 0.001; Fig. 4).

**Figure 5.**
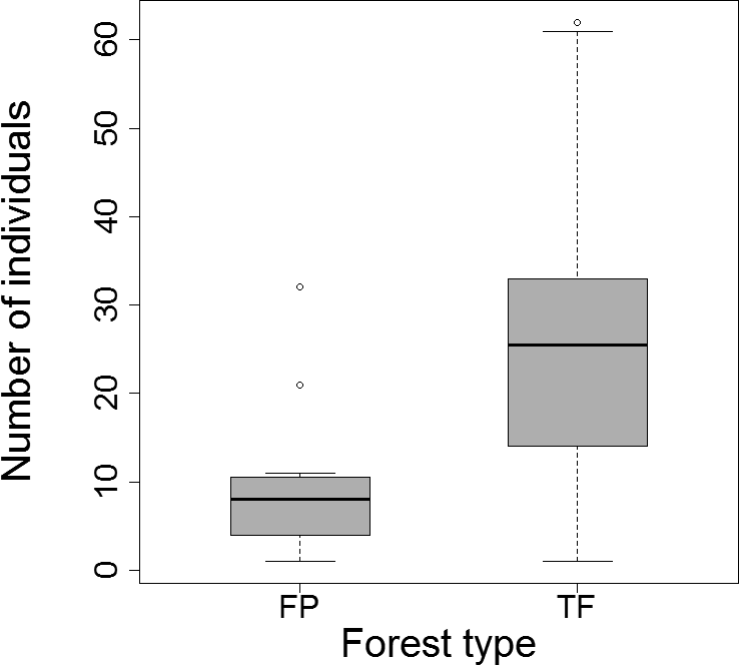
Number of Cicindelini (tiger beetles) collected from FP and TF forests. Significantly more tiger beetle individuals were collected in TF forest (P = 0.011).

**Figure D1.**
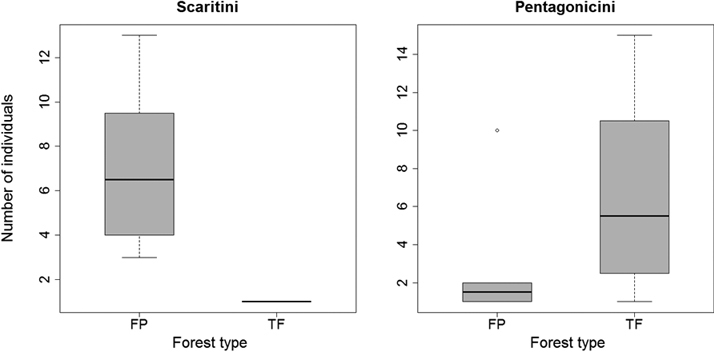
Numbers of individuals for Carabidae tribes with significant differences (P < 0.05) between FP and TF forests.

**Figure D2.**
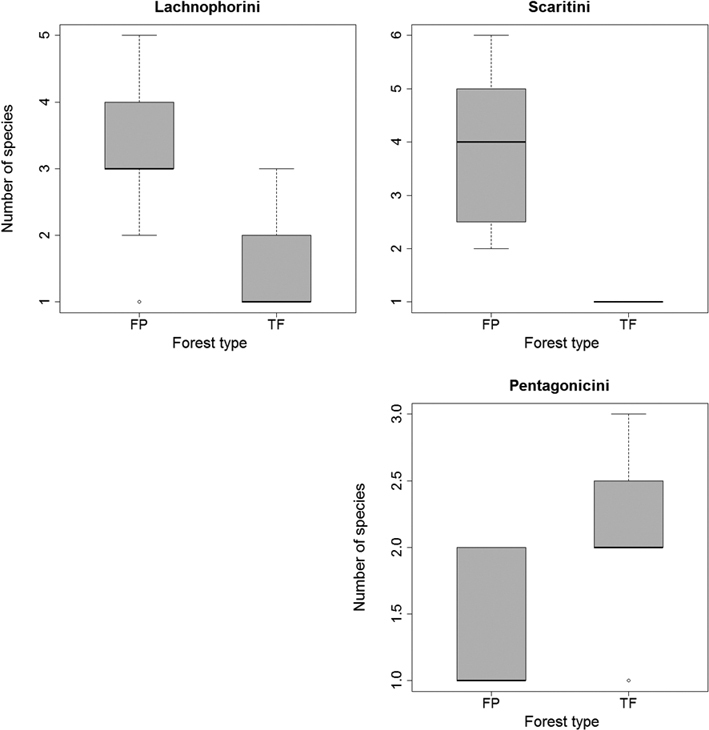
Numbers of morphospecies for Carabidae tribes with significant differences (P < 0.05) between FP and TF forests.

Carabidae of 14 tribes were represented in both habitats; however, Callistini, Collyridini, Galertini, Hiletini, Oodini, and Perigonini were collected from FP forest only with collyridines and perigones represented by single specimens (Appendix B: Table B1). The most morphospecies rich tribes for FP forest were Lebiini (14.6% of total S in FP forest), Scaritini (14.6%) and Lachnophorini (12.5%), followed by Harpalini and Bembidiini (both with 11.5%). The most morphospecies rich tribe for TF forest, by a large margin, was Lebiini (35.4% of total S in TF forest), followed by Harpalini (10.1%), Bembidiini (10.1%), and Pterostichini (8.9%). Four morphospecies of Cicindelini accounted for 35% (n = 438) of the total number of individuals in the pooled sample from both habitats, but significantly more Cicindelini were sampled in TF than FP forest (W = 24, P = 0.011; Fig. 5). Analyses for carabid tribes occurring in both forest types showed the mean number of individuals for Scaritini was significantly higher in FP forest (t = 6.5, df = 11, P < 0.001) but significantly lower for Pentagonicini (W = 14, P = 0.040; Appendix D: Fig. D1). Mean morphospecies number per site in FP forest was significantly higher for Lachnophorini (W = 64, P = 0.008) and Scaritini (t = 7.3, df = 11, P < 0.001) but significantly lower for Pentagonicini (W = 15, P = 0.036; Appendix D: Fig. D2).

**Figure C1.**
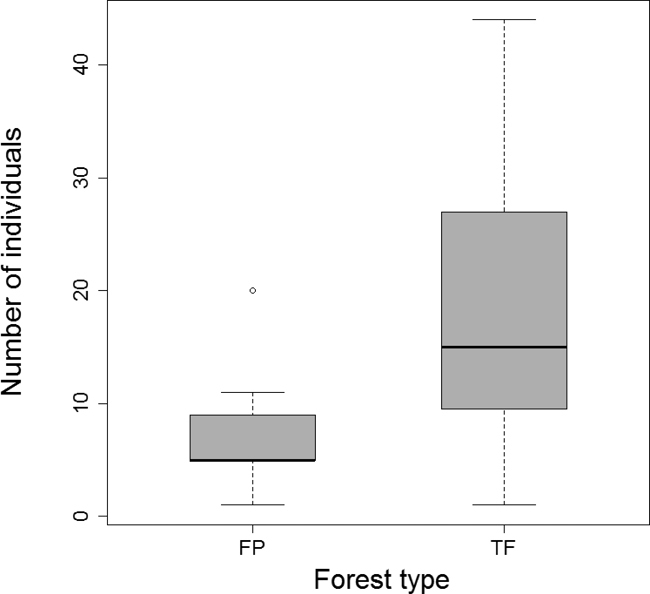
Number of individuals for the morphospecies cicindelid *Pentacomia* species b (Pentb) for FP and TF forests (P = 0.02).

The cicindelid *Pentacomia* species Pentb was the only ‘dominant’ morphospecies in the overall sample, accounting for 23.3% of individuals sampled (n = 292) and collected at 20 of the 24 sampling sites, including all 12 TF sites. The number of cicindelid *Pentacomia* morphospecies individuals collected in TF forest was significantly higher than in FP forest (W = 20.5, N = 21, P = 0.02; Appendix C: Fig. C1) and it also accounted for a higher percentage (32.7% vs. 11.7%) of the sample than in the FP forest. Eighteen morphospecies were classified as ‘common’ (12.6% of the total S) and 125 morphospecies as ‘rare’ (87.4% of total S). Individuals classified as common made up 48.9% (n = 614) while rare individuals contributed 27.8% (n = 349) of the total number of individuals collected. Among rare morphospecies, 63 were represented by a single individual (singletons) and 22 morphospecies by two individuals (doubletons). Of the total number of morphospecies collected, 43.8% were singletons and 15.3% doubletons. There were no differences between forest types in mean number of individuals collected for common or rare morphospecies.

**Figure C2.**
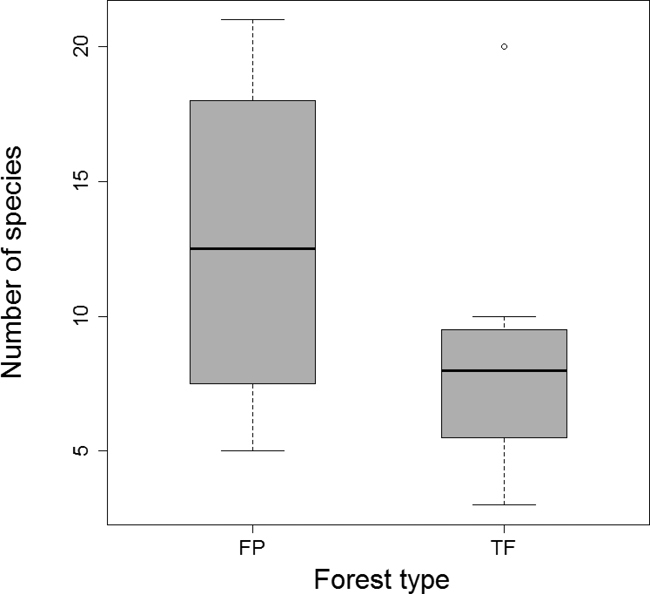
Number of rare morphospecies for the FP and TF forest (P = 0.04).

**Figure C3.**
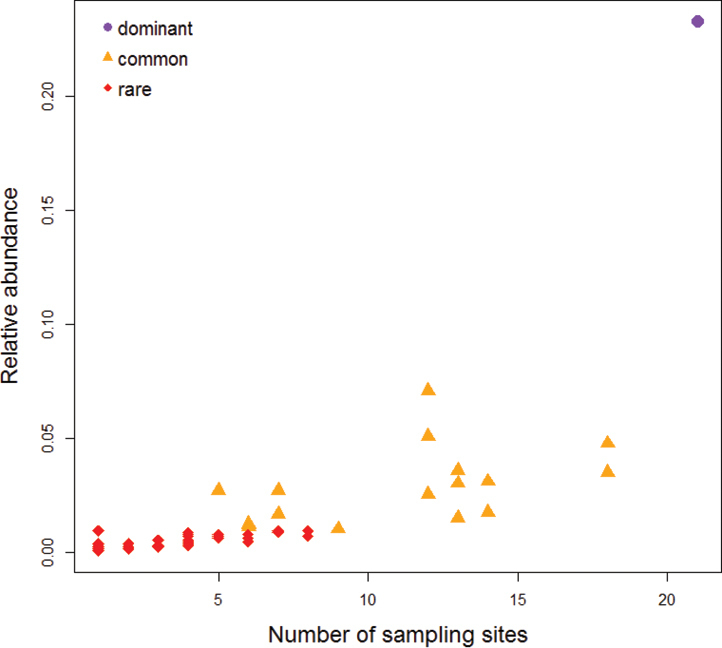
Morphospecies relative abundance by the number of sampling sites at which they were present. Point markers represent the 143 morphospecies coded according the three rarity categories: ‘dominant’ (circle), ‘common’ (triangles) and ‘rare’ (diamonds). Morphospecies classified as ‘dominant’ occurred at a higher number of sampling sites than ‘common’ morphospecies. ‘Common’ morphospecies occurred at a higher number of sampling sites than ‘rare’ morphospecies (P < 0.001).

Rare morphospecies contributed the largest proportion of total morphospecies richness for both forest types (80.2% for FP forest and 78.5% for TF forest), although the mean number of rare morphospecies per site was significantly higher for FP forest (13 ± 1.5) than TF forest (8 ± 1.3) (t = 2.2, df = 22, P = 0.04; Appendix C: Fig. C2). Numbers of common morphospecies per site did not differ between forest types (8 ± 0.5). All but two common morphospecies (90% of total common S) occurred in both forest types while only 15 rare morphospecies (12% of total rare S) occurred in both forest types. As expected, the dominant morphospecies occurred at a higher number of sampling sites than common morphospecies and common morphospecies occurred at a higher number of sampling sites than rare morphospecies (χ^2^ = 291.2, df = 2, P <0.001; Appendix C: Fig. C3).

Overall, the ‘dominant’ morphospecies, cicindelid Pentb, and the 18 common morphospecies contributed 72.2% of the total number of individuals collected and represented seven carabid tribes. However, distribution of the 19 morphospecies within these seven tribes represented differed significantly from expected values based on the total number of morphospecies collected in each tribe (chi-square χ^2^ = 33.6, df = 6, P < 0.001; Appendix C: Table C1). In contrast, the distribution of 124 rare morphospecies within 19 tribes was not significantly different than expected.

**Figure 6.**
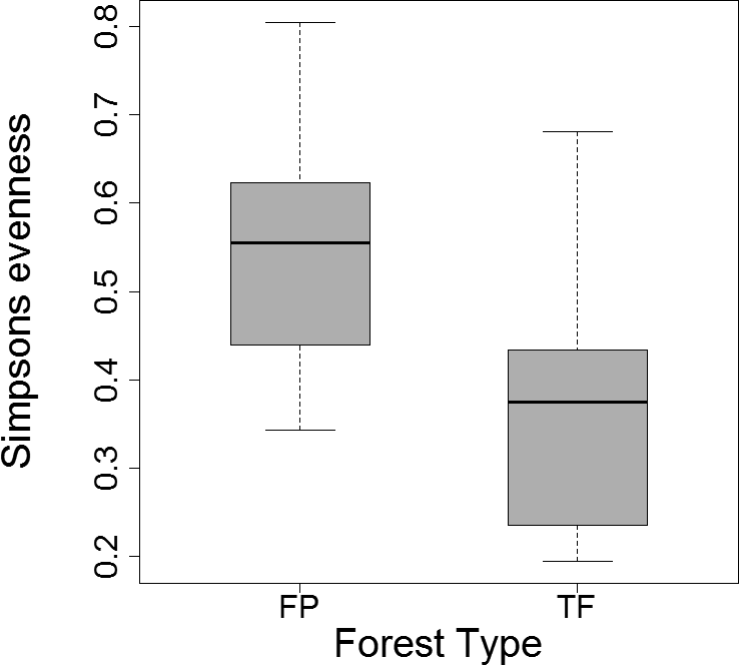
Simpson’s evenness index (E_1/D_) for species assemblages were significantly more even from FP than TF forests (P = 0.008).

**Figure 7.**
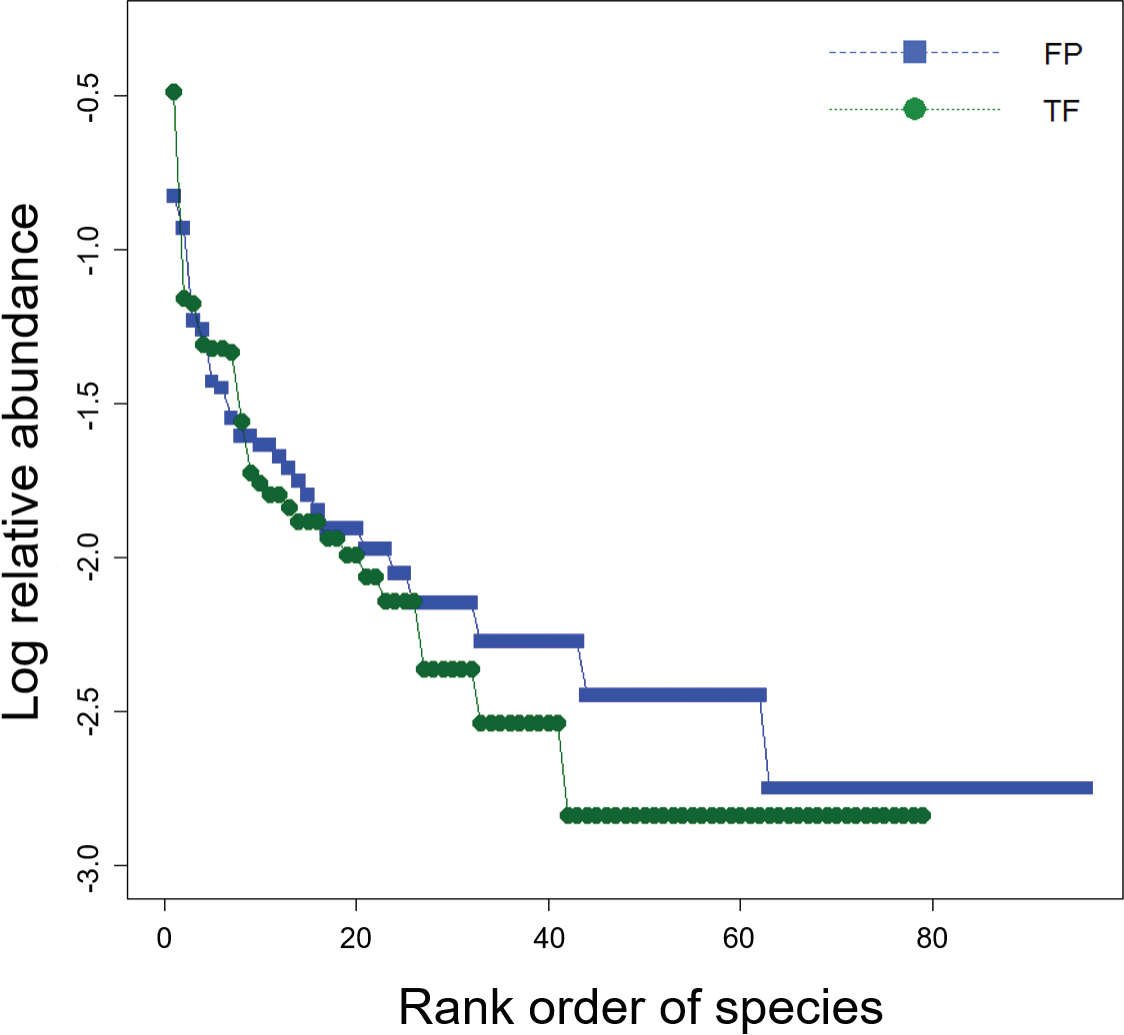
Rank abundance distribution curves for FP (blue squares) and TF (green circles) forests.

Simpson’s evenness index (E_1/D_) indicated that morphospecies assemblages were significantly more even in FP than in TF forests (t = 2.9, df = 22, P = 0.008; Fig. 6). Although the rank abundance distribution curves for both habitats (Fig. 7) have long ‘tails’ of rare morphospecies, the curve for FP forest underscores the increased evenness of morphospecies assemblages in FP compared to TF forests. In contrast, dominance of cicindelid Pentb, the most abundant morphospecies in both habitats, is most evident in the TF abundance curve. The abundance distribution curve for FP forest is longer, reflecting the increased morphospecies richness of this forest. The greater contribution of rare morphospecies lowered the slope of the curve beyond rank 25, near the division between common and rare morphospecies. More morphospecies were represented by three or four individuals in FP compared to TF forests (n = 18 vs. 6) as well as more doubleton morphospecies in FP (n = 19 vs. 9). In contrast, there were slightly more singletons collected in TF forest than in FP forest (n = 33 vs. 30).

**Figure 8.**
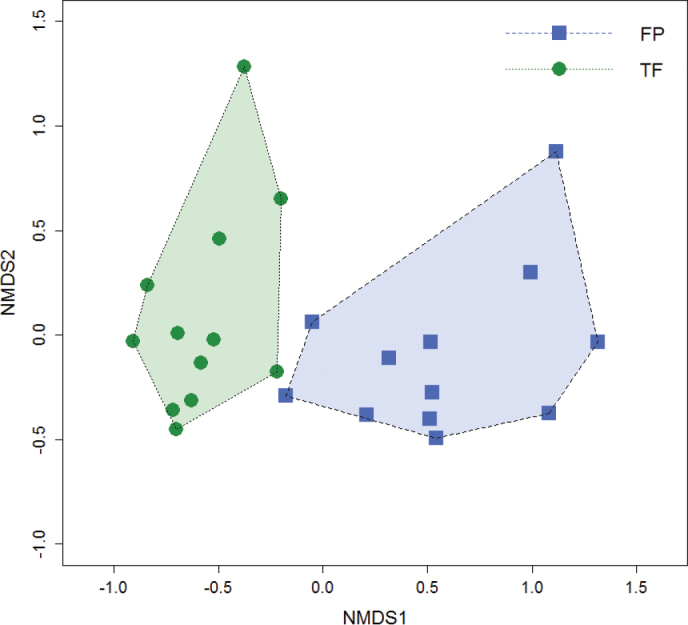
Non-metric multidimensional scaling (NMDS) ordination using Bray-Curtis dissimilarity for carabid morphospecies assemblages from FP and TF forests (stress = 13.7, k = 2). Each data point represents one of 24 sampling sites, with blue squares representing FP forest sites and green circles representing TF forest sites. Morphospecies assemblages were significantly different between FP and TF (P < 0.001).

NMDS ordination demonstrated clear separation between FP and TF morphospecies assemblages with no overlap in their respective multivariate polygons (Fig. 8; stress = 13.7). The greatest divergence was along axis 1, with FP sites aggregated on the positive side and TF sites on the negative side of the axis. The mean within group Bray-Curtis dissimilarity was 0.68 for FP forest and 0.65 for TF forest while the between group dissimilarity value was 0.81, with significant differences between FP and TF morphospecies assemblages (F = 6.64, df = 1, P < 0.001). The area of the FP polygon (1.18) was larger than the TF polygon (0.72) but there was no difference in dispersion within ordination space between habitats. The two forest types shared only 32 of the 143 total morphospecies (22%) and 21 of 54 total genera (38%) represented in the samples. When singletons were eliminated from the comparison, FP and TF shared 40% of morphospecies and 51% of genera underscoring the importance of rare morphospecies to the differences. Both the low number of shared morphospecies and high between group mean Bray-Curtis dissimilarity value are reflected in the ordination biplot (Fig. 8). To determine the influence of morphospecies abundance on the NMDS, a second ordination analysis was conducted using presence/absence data and Jaccard dissimilarity values. The resulting patterns are similar to the results using the abundance data (see Appendix E for additional details).

Several morphospecies had significant indicator values (> 0.7) for either FP or TF forests (Table 2). Most of these morphospecies had high specificity values (A) for one forest type (mean = 0.96) while fidelity values were relatively lower (mean = 0.61). Interestingly, different morphospecies of the genera *Paratachys* (Bembidiini) and *Peruphorticus* (Lachnophorini) were significant indicators for each of the two forest types. Several taxa within the Scaritini (*Nyctosyles* and *Clivina*) and a lachnophorine species of *Eucaerus* (Lachnophorini) were significant indicators of FP forest while morphospecies from within Pentagonicini (*Pentagonica*) were significantly associated with TF forest.

**Table 2.** List of the characteristic carabid species with significant indicator values for FP and TF forests using the IndVal statistic of ISA by Dufrêne and Legendre (1997). ‘A’ measures the specificity of a given species using relative abundance within and among sampling sites for a forest type and ‘B’ measures fidelity though relative frequency within the sampling sites of each forest type. Adjusted p-values (p.adj holm) for multiple testing correspond to experiment-level conclusions.

Morphospecies code	Tribe: Genus	A	B	IndVal index	p	p.adj (holm)
*FP*						
Clivb	Scaritini: Clivina	0.969	0.917	0.942	0.001	0.025*
Perua	Lachnophorini: Peruphorticus	0.94	0.917	0.93	0.001	0.025*
Nyctb	Scaritini: Nyctosyles	1	0.583	0.764	0.005	0.110
Nycta	Scaritini: Nyctosyles	0.917	0.583	0.731	0.021	0.378
Eucaa	Lachnophorini: Eucaerus	1	0.500	0.707	0.013	0.247
Parac	Bembidiini: Paratachys	1	0.500	0.707	0.012	0.24
*TF*						
Pentgd	Pentagonicini: Pentagonica	0.895	0.833	0.863	0.001	0.025*
Lebih	Lebiini: Lebia	1	0.500	0.707	0.01	0.21
Perua_2	Lachnophorini: Peruphorticus	0.971	0.500	0.697	0.031	0.527
Pentga	Pentagonicini: Pentagonica	0.917	0.500	0.677	0.039	0.72
Parae	Bembidiini: Paratachys	1	0.417	0.645	0.045	0.72

## Discussion

### Faunal differences between FP and TF habitats

Results from our study indicate that richness, diversity, and morphospecies composition of understory Carabidae from eastern Ecuadorian lowland rainforests are influenced by forest type. As expected, the simple comparison between hand and FIT collections presented here suggests the two methods sampled different portions of the carabid fauna in each forest type, similar to results reported in other tropical arthropod studies (Gadagkar et al. 1990; Kitching et al. 2001; Missa et al. 2009; Riley et al. 2016). Employing both collection techniques sampled a greater portion of the overall carabid community than either collection technique alone, providing a more comprehensive sample of Carabidae in FP and TF forests.

The number of individual carabids collected in the understories of FP and TF forests did not differ significantly. In contrast, Lamarre et al. (2016) found Carabidae, including cicindelids, were strikingly more abundant in seasonally flooded forests than in TF forests in French Guiana and Peru, with ~ 91% of cicindelids and 75% of all remaining carabids collected in flooded forests. This disparity may be due to several factors, including differences between the studies in species composition, sampling protocols, sample completeness, and the time period and duration of sampling periods. Other studies in Central Amazonia found higher tiger beetle richness in TF forests (Adis et al. 1998; Zerm et al. 2001; Adis and Junk 2002). In our study, all four Cicindelini species occurred in both FP and TF forests but significantly more cicindelid individuals were collected from TF forest. Some studies from other parts of the Amazon reported higher tiger beetle abundance in TF forests (Zerm et al. 2001; Adis and Junk 2002), in contrast higher abundance has also been reported in FP forests (Pearson and Derr 1986; Lamarre et al. 2016). Thus, the data are insufficient to suggest a general abundance pattern for Cicindelini occurring in these two Amazonian forest types.

Studies of invertebrate assemblages as a whole have generally found lower abundances within the understory of FP forests compared to TF forests (Pearson and Derr 1986; Mertl et al. 2009; Lamarre et al. 2016). Since the flooding cycle causes seasonal movements and shifts in the biotic communities of riparian areas, the time of year will likely affect the relative number of individuals occurring in the understories of these two forest types (Irmler 1979a; Adis 1981; Erwin and Adis 1982; Adis and Junk 2002). Abundance patterns of adult Carabidae vary in space and time, and are affected by factors such as flooding duration, frequency, and intensity (Adis 1981; Bonn 2000; Bonn et al. 2002; Lambeets et al. 2008, 2009).

Although mean morphospecies richness differed only marginally (P = 0.06), all other measures of richness and diversity were significantly higher in FP forest compared to TF forest, corroborating results of some previous studies (e.g., Erwin and Adis 1982; Pearson 1984; Pearson and Derr 1986). The higher species richness and diversity of FP forest seems to reflect the higher number of rare morphospecies sampled there than in TF forest. Increases in Carabidae richness after flooding events has been recorded for Central Amazonian forests (Adis 1981), as well as for temperate forests (Bonn 2000; Ellis et al. 2001; Bonn et al. 2002; Lambeets et al. 2008, 2009). After inundation, the litter layer and ground vegetation of FP habitats at TBS are covered by a thin layer of mud and sediments (pers. obs.; Mertl et al. 2009). This may result in higher nutrient content or resource availability when waters recede that promotes features like higher prey abundance that are attractive to particular carabid species (Irmler 1979a, b; Duivenvoorden and Lips 1995; Bonn 2000; Adis and Junk 2002; Haugaasen and Peres 2005; Adis et al. 2010). Disturbance may increase the number of unoccupied microhabitats in post-flood areas, generating a reciprocal increase alpha diversity over time (Salo et al. 1986; Erwin 1991b; Kalliola et al. 1991; Zerm and Adis 2001a; Wittmann et al. 2006). Lastly, the disturbance of flooding could also moderate competitive exclusion, resulting in higher diversity in floodplain forests by promoting higher levels of coexistence (Levin and Paine 1974; Connell 1978; Erwin and Adis 1982; Zerm and Adis 2001a, b).

Differences in carabid beetle richness and diversity patterns among localities within Amazonia may reflect variation among sites, particularly because erosion, deposition and changes in river channels continuously drive spatial and temporal changes in flooded forests (Kalliola et al. 1991; Junk 2000). Faunal richness and diversity have been strongly tied to length, frequency and severity of flooding events (Bonn et al. 2002), and these vary among Amazonian rivers depending on factors such as flooding cycles, topography and weather patterns (Junk et al. 1989; Junk 2000; Tuomisto et al. 2003; Adis et al. 2010; Junk and Piedade 2010). Temporarily flooded forests with less severe and frequent flooding regimes, such as those near TBS, will likely show different patterns in richness and diversity than those subjected to more intense flooding regimes.

Erwin (1979) and Erwin and Adis (1982) previously suggested that a large proportion of carabid species inhabit riverine and wetland habitats in Amazonia. A long history of carabid evolution in tropical wetland habitats (Erwin 1979, 1985), is consistent with adaptation of many species to changing environmental conditions in riparian zones (Adis 1982). Carabid traits that promote persistence during unfavorable periods of the flooding cycle include: synchrony of life cycles with patterns of disturbance, reproductive dormancy, and submersion tolerance (Erwin 1979; Adis 1982; Erwin and Adis 1982; Paarmann et al. 1982; Erwin 1985; Adis et al. 1986; Amorim et al. 1997; Zerm and Adis 2001b, 2003). Although Amazonian wetland and floodplain habitats have experienced repeated reductions and expansions through changes in paleoclimate (e.g., Cheng et al. 2013), they have doubtlessly continued to be available supporting persistence and radiation of species with adaptions to flooding cycles (Erwin and Adis 1982; Kubitzki 1989; Irion et al. 1995, 1997, 2010; Müller et al. 1995; Haffer and Prance 2001; Adis and Junk 2002; Hoorn et al. 2010; Wittmann et al. 2010).

Carabids have patchy distributions, particularly in tropical rainforests, making the average abundance of many species appear low and requiring higher collection effort to obtain a representative sample of the fauna (Erwin 1979; Paarmann et al. 2002; Erwin et al. 2005). Sample completeness, based on the average of three nonparametric diversity estimators, was greater in FP forests (~ 74%) than TF forests (~ 55%). This, in turn, influences the observed richness and diversity values for both forest types. A possible contribution to the lower observed richness and sample completeness in TF forests was the lower efficiency of hand sampling at TF sites compared to FP sites. While the exact reason for the lower efficiency is unknown, possible explanations include an increased vertical stratification of Carabidae in TF forest and higher aggregation of carabid spatial distributions in FP forests. Additionally, non-cicindelid carabid individuals collected from TF sites were, on average, smaller relative to those collected at FP sites (Fig. 4) and such differences may also negatively influence the collectability of individuals in TF forests (see Riley et al. 2016 for further discussion).

Based on extrapolation of rarefaction curves, many morphospecies remained uncollected in TF forests. Although the three diversity estimators we employed gave somewhat different answers, the differences between FP and TF assemblages were relatively small. Nonetheless, we predict that the observed difference in morphospecies richness between FP and TF sites will decrease with greater sampling effort. Within Amazonia, TF forests are more extensive than FP forests, and thus given standard expectations of species-area relationships, richness and diversity of TF forests are expected to be greater than for FP forests across the region (Rosenzweig 1995; ter Steege et al. 2000; Pitman et al. 2001; Burnham 2002; Tuomisto et al. 2003; Myster 2014). Compared to TF forests, FP forests are limited to narrow areas along the banks of rivers and lakes and, at the landscape level, are more fragmented (ter Steege et al. 2000; Pitman et al. 2001).

Differences between FP and TF forests occurred not only at the morphospecies level but also at the tribal level. There were higher numbers of Scaritini and Lachnophorini individuals and/or morphospecies in FP forest and higher numbers of Pentagonicini and Cicindelini individuals and/or morphospecies in TF forest. Although life histories are unknown for the vast majority of Amazonian carabid species, general life history information is known for many of the sampled genera and tribes. Species within Scaritini and Lachnophorini are typically ground-dwelling whereas those of the Pentagonicini and Cicindelini are more likely to be sampled on vegetation (per. obs.; Erwin et al. 2012). Many Lachnophorini and Scaritini species occur in wetter habitats which may explain why they were collected in greater numbers and diversity in FP forests in this study (Erwin et al. 2012; Erwin and Zamorano 2014). Indicator species analysis (ISA) showed two genera, *Peruphorticus* (Lachnophorini) and *Paratachys* (Bembidiini), had one morphospecies characteristic of FP and another morphospecies characteristic of TF forest. Differences in microhabitat associations among tribes and genera likely attributed to observed differences in tribal-level richness and abundances, as well as variations in morphospecies composition between forest types.

### Community-level differences between FP and TF forests

Rank abundance curves have rarely, if ever, been reported for tropical Carabidae. In temperate and boreal forests, carabid communities typically have a few dominant species with the majority of species being rare (Belaoussoff et al. 2003), similar to the assemblages for FP and TF forests in this study. Dominant and common morphospecies comprised 72.2% of the total number of individuals collected but only 13.3% of the total observed morphospecies. As with many tropical insect assemblages, the contribution of rare species was especially high (Novotný and Basset 2000), with rarer species accounting for a greater proportion of the carabid assemblages than in temperate communities. In our study, singletons contributed slightly less than half of the overall morphospecies richness (43.8%), which is more than a canopy fogging study in a nearby Ecuadorian TF forest where 28.6% were singletons (Lucky et al. 2002).

Chi-square analyses examining rarity categories among carabid tribes showed abundant morphospecies were significantly more likely to be within the Bembidiini, Cicindelini, Lachnophorini, Loxandrini, and Pentagonicini, with fewer morphospecies of Lebiini collected than expected. Although Lebiini was by far the most morphospecies rich tribe (S = 35), only two morphospecies were classified as common while the remaining 33 morphospecies occurred at low numbers. In contrast, all four of the Cicindelini morphospecies were classified as abundant. Common morphospecies were sampled from a higher number of sampling sites within the study area, suggesting that they have wider spatial distributions than less frequently encountered morphospecies. This agrees with previously reported positive relationships between local population abundance and the occurrence at a greater proportion of sample sites (Hanski 1982; Brown 1984; Gaston and Lawton 1988; Niemelä and Spence 1994).

Analyses indicate distinct carabid morphospecies assemblages occur in FP and TF forests, and this is further supported by the number of characteristic morphospecies for each forest type. In our study, only 32 of 143 morphospecies were collected in both forest types, suggesting FP forests maintain unique assemblages compared to neighboring non-flooded TF forests. Near Manaus, Brazil, Zerm et al. (2001) reported only two of 25 tiger beetle species occurring at both FP and TF forest sites. Unique species assemblages for FP and TF forests has been recorded for tropical vegetation (ter Steege et al. 2000; Bredin et al. 2020), birds (Remsen and Parker 1983; van Lieshout et al. 2016), understory arthropods (Adis and Junk 2002; Lamarre et al. 2016), canopy arthropods (Erwin 1983b; Adis et al. 2010), spiders (Höffer 1997), and understory tiger beetles (Zerm et al. 2001). Differences in species assemblages may relate to factors such as flooding regime adaptions, differences in plant composition, forest structure, and resource availability between FP and TF forests. The spatial and temporal distribution of tiger beetle guilds have been shown to be influenced by specific microhabitats within a given habitat (such as open areas, forest type, etc.) (Zerm and Adis 2001a; Zerm et al. 2001; Adis and Junk 2002). Lamarre et al. (2016) reported significant associations for seasonally FP forest or TF forests for several arthropod families, with carabid beetles demonstrating a particularly strong association with seasonally flooded forests.

### Specialization of life in FP forests

Since flooded forests oscillate between aquatic and terrestrial phases and there is high variability in flooding events among rivers, it is difficult to adequately measure the overall biodiversity and community composition of flooded forests as a whole (Junk et al. 1989; Junk 2000). Floodplain communities are a mix of migrants, visiting species and residents, the latter likely with highly specialized adaptions for inundation events (Adis 1997; Junk 1997; Adis and Junk 2002). Thus, presence of both resident and migrant species in FP forest may have contributed to the higher observed richness, diversity, and the higher number of rare morphospecies collected in the FP forest. Carabids seeking refuge from flooding events may move into the canopy or sub-canopy strata via tree trunks (Irmler 1973, 1979a; Adis 1981; Adis and Messner 1997), move horizontally following the waterline (Irmler 1979a; Adis and Messner 1997; Zerm and Adis 2003), or fly to non-flooded uplands (Irmler 1973; Adis 1982; Erwin and Adis 1982; Adis et al. 1986). Thus, selection for escape from flooding may help explain why the majority of carabid beetles living in tropical forests are fully winged and presumably capable of flight (Erwin 1979). Other specializations to flooding regimes have also been reported for adults and possibly larvae, including retreat to subsoil layers (e.g., a flightless *Stratiotes* species), aestivation and submersion tolerance (Adis 1982, 1997; Adis et al. 1986; Rothenbucher and Schaefer 2006; Lambeets et al. 2009).

Terborgh and Andresen (1998) suggest that vegetation of floodplain forests may be more closely allied taxonomically to adjacent non-flooded forests rather than to other flooded forests. Conversely, specific adaptions to life in flooded forests could promote convergence in floodplain community composition, even at broad geographic scales (e.g., Lamarre et al. 2016). In the present study, morphospecies in two genera (*Paratachys* and *Peruphorticus*) were significantly associated with either FP or TF forests suggesting that there are evolutionary pressures to specialize with respect to forest type. Or, it could be a combination of both factors. For Amazonian frogs and mammals, Gascon et al. (2000) showed habitat type (FP vs. TF forest) and geographic distance were the most significant predictors of community similarity.

## Conclusions

Even though conclusions must be tempered by the spatio-temporal scale of our sampling, this is one of the few studies that compares carabid assemblages between major Amazonian forest types. Our work underscores that FP assemblages differ significantly from those in TF forest in this region of western Ecuador, and that additional sampling at TBS may better define the overlap in species between the two forest types. Activity of many Amazonian Carabidae is seasonal, particularly in habitats that are flooded periodically (Paarmann 1986; Pearson and Derr 1986; Adis and Junk 2002; Erwin et al. 2005; Cajaiba et al. 2018), and the dynamics of these assemblages have not been well studied. Clearly, a more comprehensive representation of overall Carabidae richness, diversity and species assemblages requires sampling that extends throughout the year (Pearson and Derr 1986; Lucky et al. 2002; Erwin et al. 2005), as well as the use of multiple collection techniques (e.g., hand sampling, FITs, canopy fogging, etc.) (Gadagkar et al. 1990; Kitching et al. 2001; Missa et al. 2009). Focused sampling should occur before and after flooding events (Junk 2000) to better understand the dynamics of FP assemblages. A paired-site study design at a larger geographic scale within Amazonia would elucidate patterns in carabid richness, diversity and species composition in FP or TF forests.

Our research targeted carabids using ground and understory habitats, but to understand the overall patterns in diversity and structure of carabid species assemblages within Yasuní forests, all forest strata should be included in community-level analyses (Basset et al. 2001, 2003; Chung 2004; Charles and Basset 2005). Objectives of such work must also include assessment of the forest canopy fauna since tropical canopies appear to be even more species rich than understory habitats (Erwin 1982; Charles and Basset 2005; Erwin et al. 2005). Anthropogenic factors, particularly oil exploration and extraction and climate change are now driving broad changes to the fauna of these megadiverse areas (Finer et al. 2009; Bass et al. 2010; Kirichenko-Babko et al. 2020). Thus, gaining increased understanding of rainforest insect assemblages like those at Yasuní is critical to assess and ameliorate the impacts, and to conserve the natural fauna of these unique habitats.

## Figures and Tables

**Figure A1.**
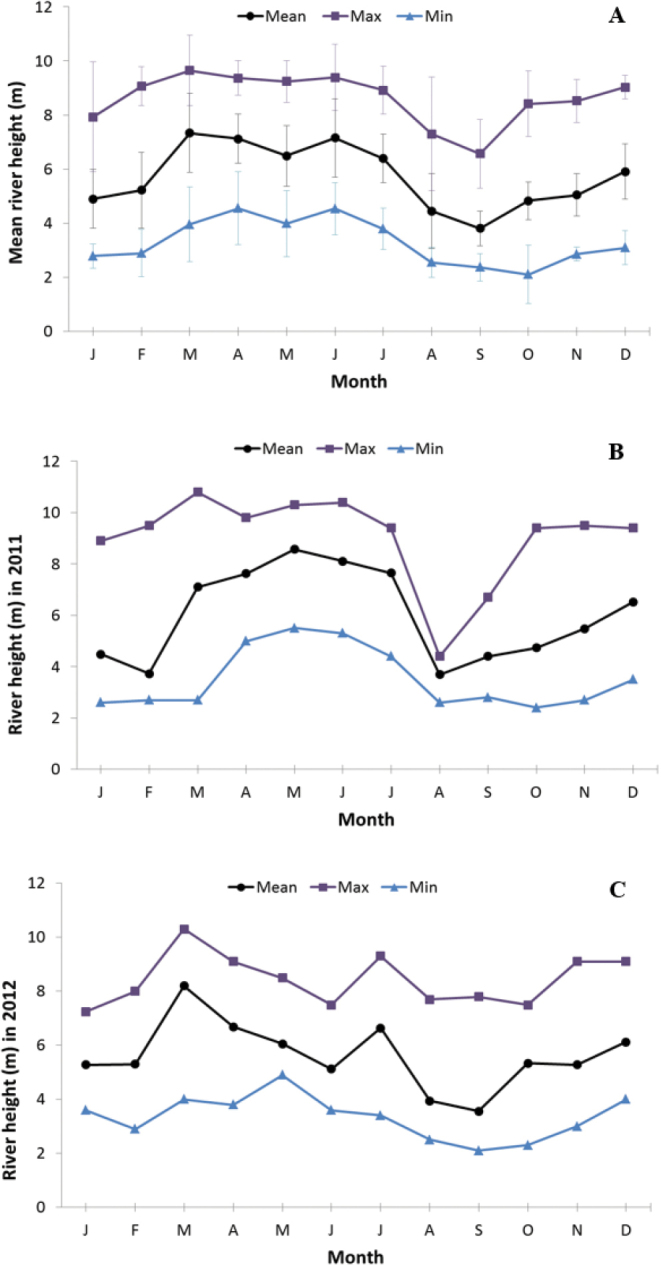
Water height for the Tiputini River at Tiputini Biodiversity Station, Ecuador. (**A**) Monthly values represent overall mean river height and the mean maxima and minima based on data from 2009–2014 (**B**) three years before and after the sampling period for this study. Monthly river height values during the sampling periods, 2011 (**B**) and 2012 (**C**) for mean river height in addition to water height maxima and minima.

**Figure E1.**
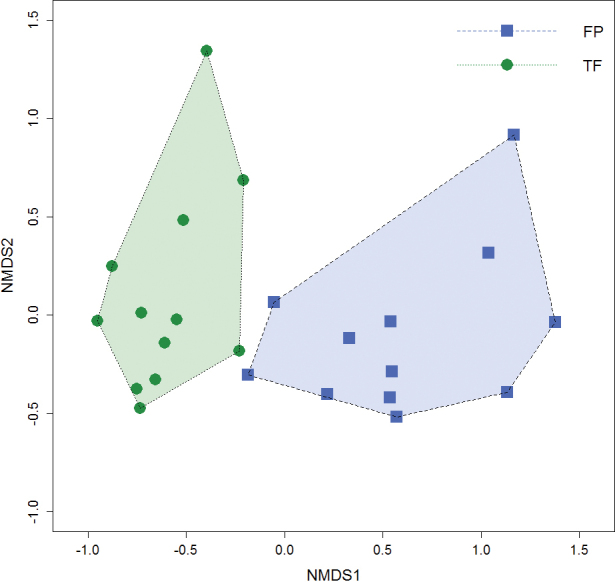
Non-metric multidimensional scaling (NMDS) ordination based on Jaccard dissimilarity values (presence/absence data) of Carabidae morphospecies assemblages for FP and TF forests (stress = 13.7, k = 2). Each data point represents one of the 24 sampling sites. Significant differences (P < 0.001) in morphospecies assemblages occurred between FP and TF.

## Appendix B

**Table B1.** Numbers of carabid individuals collected in FP and TF forests, as well as combined totals, are shown in the table below. The summed totals of individuals for each tribe and morphospecies/species are shown in bold, along with the identification code used for each morphospecies. If a species has been scientifically described and given a taxonomic name, it is noted to the left of the identification code. Lastly, ABL (mm) for singletons or mean ABL if > 3 specimens collected for each morphospecies or species. In the length column, “NA” signifies no intact specimens were collected so measurements could not be taken.

	Carabidae taxa	FP	TF	Total	ABL (mm)
	** Bembidiini **	**65**	**42**	**107**	
	* Erwinana *				
	Erwia	1		1	3.1
	Erwib		1	1	3.1
	* Geballusa *				
	Gebasp	2	6	8	3.6
	* Meotachys *				
	Meotsp	2		2	2.3
	* Micratopus *				
	Micrsp	4		4	2.5
	* Mioptachys *				
	Miopsp		1	1	NA
	*New Genus 1*				
	NG1a	3		3	1.4
	NG1b		1	1	1.8
	*New Genus 2*				
	NG2sp	2		2	2.5
	* Paratachys *				
	Paraa	33	11	44	1.9
	Parab	2	13	15	2.7
	Parac	14		14	2.5
	Parad	1		1	NA
	Parae		9	9	2.5
	* Polyderis *				
	Polysp	1		1	1.4
	** Callistini **	**6**		**6**	
	* Dercylus *				
	Derca	4		4	13.0
	Dercb	2		2	13.6
	** Calophaenini **	**2**	**4**	**6**	
	* Calophaena *				
	Caloa	2		2	7.1
	Calob		3	3	8.9
	Caloc		1	1	7.3
	** Cicindelini **	**109**	**329**	**438**	
	* Odontocheila *				
**Odontocheila cayenensis* Lam.	Odona*	14	46	60	16.1
	Odonb	16	48	64	11.8
	* Pentacomia *				
	Penta	13	9	22	11.9
	Pentb	66	226	292	9.9
	** Collyridini **	**1**		**1**	
	* Ctenostoma *				
	Ctensp	1		1	NA
	** Galeritini **	**6**		**6**	
	* Galerita *				
	Galea	2		2	17.8
	Galeb	2		2	17.6
	Galec	2		2	17.2
	** Harpalini **	**36**	**24**	**60**	
	* Notiobia *				
	Notia	7	3	10	10.3
	Notia_2	2	1	3	9.9
	Notia_3	2	7	9	9.8
	Notib	8	3	11	8.4
	Notib_2	1		1	8.1
	Notib_3	1		1	8.2
	Notib_4	1		1	9.2
	Notic	1		1	13.5
	* Selenophorus *				
	Selea	3	5	8	6.5
	Seleb		2	2	5.7
	Selee	9	2	11	7.1
	Selee_6	1		1	6.4
	Selef		1	1	5.5
	** Helluonini **	**6**	**3**	**9**	
	* Helluobrochus *				
	Hellub_a		1	1	13.7
	Hellub_b		1	1	13.5
	* Helluomorpha *				
	Hellum	1		1	NA
	* Helluomorphoides *				
	Hella	3		3	17.3
	Hellb		1	1	13.9
	Hellc	2		2	24.7
	** Hiletini **	**5**		**5**	
	* Eucamaragnathus *				
**Eucamaragnathus batesi* Chaudoir	Eucabat*	5		5	9.2
	** Lachnophorini **	**129**	**41**	**170**	
	* Amphithasus *				
	Ampha	2		2	6.0
	Amphb	1		1	4.7
	* Eucaerus *				
	Eucaa	6		6	3.9
	Eucaa_2	3		3	3.8
	Eucab	1		1	3.5
	* Peruphorticus *				
	Perua	84	5	89	6.3
	Perua_2	1	33	34	5.3
	Perua_3	13	3	16	6.2
	Perub	7		7	4.3
	Perub_2	1		1	4.4
	* Pseudophorticus *				
	Pseua	6		6	6.1
	Pseub	4		4	5.3
	** Lebiini **	**30**	**90**	**120**	
	* Apenes *				
	Apena		1	1	5.6
	Apena_2	1	1	2	7.1
	Apena_3	2		2	5.5
	Apenb	1	2	3	6.2
	Apenb_2		1	1	6.8
	Apenb_3a		1	1	7.6
	Apenb_3b	1		1	7.9
	Apenb_3c		2	2	6.7
	Apenb_4		1	1	5.7
	Apenb_5		1	1	7.3
	Apenbb		1	1	5.7
	Apenc		1	1	7.9
	Apenc_2		1	1	8.6
	Apenc_3		1	1	7.1
	Apene		1	1	5.7
	* Coptodera *				
	Copta		1	1	8.6
	Coptb		1	1	8.4
	* Eucheila *				
	Euchsp		1	1	4.9
	* Hyboptera *				
	Hybosp		2	2	NA
	* Lebia *				
	Lebia	3	5	8	3.4
	Lebib		1	1	4.4
	Lebic	1		1	5.8
	Lebid	2	32	34	5.5
	Lebid_5	1		1	6.0
	Lebie	7	8	15	5.0
	Lebif	1		1	3.9
	Lebig		2	2	7.5
	Lebih		10	10	4.8
	Lebij		1	1	5.3
	Lebik	4	1	5	3.8
	Lebil	2	8	10	5.1
	Lebio		1	1	3.6
	* Negrea *				
	Negrsp		1	1	3.0
	* Nemotarsus *				
	Nemosp	1		1	4.7
	* Stenognathus *				
	Stensp	3		3	9.5
	** Loxandrini **	**45**	**47**	**92**	
	* Adrimus *				
	Adrisp	1		1	8.3
	* Loxandrus *				
	Loxaa		1	1	6.6
	Loxab	20	19	39	6.3
	Loxac		3	3	7.1
	Loxae	7	6	13	6.5
	Loxae_2	2		2	6.1
	Loxae_3	10	9	19	6.7
	Loxae_4		7	7	5.9
	Loxae_6	2		2	7.3
	Loxaf		1	1	5.1
	Loxag		1	1	6.7
	* Stolonis *				
	Stolsp	3		3	6.2
	** Odacanthini **	**8**	**2**	**10**	
	* Colliuris *				
	Colla	3		3	7.8
	Collb		1	1	9.6
	Collc	4		4	6.0
	Collc_2		1	1	6.0
	Colld	1		1	8.5
	** Oodini **	**6**		**6**	
	* Macroprotus *				
	Macrsp	5		5	10.4
	* Oodinus *				
* most likely *O. amazonas* Chaudoir, *O. limbellus* Chaudoir, or *O. piceus* Motschulsky	Oodisp*	1		1	4.4
	** Pentagonicini **	**17**	**78**	**95**	
	* Pentagonica *				
	Pentga	1	11	12	4.6
	Pentgb	12	33	45	5.5
	Pentgd	4	34	38	4.3
	** Perigonini **	**1**		**1**	
	* Perigona *				
	Perisp	1		1	5.5
	** Platynini **	**6**	**3**	**9**	
	* Glyptolenus *				
	Glypsp	6	3	9	6.8
	** Pterostichini **	**1**	**23**	**24**	
	* Abaris *				
	Abara		12	12	5.2
	Abarb		5	5	6.1
	* Haplobothynus *				
	Hapla		2	2	8.2
	Haplb	1		1	8.8
	* Pseudabarys *				
	Pseuabsp		1	1	7.2
	* Trichonillia *				
	Trica		2	2	14.5
	Tricb		1	1	15.2
	** Scaritini **	**84**	**3**	**87**	
	* Ardistomis *				
	Ardisp	1		1	9.2
	* Camptidius *				
	Camptisp	1		1	3.5
	* Camptodontus *				
	Camptosp		1	1	13.2
	* Clivina *				
	Cliva	2		2	5.4
	Clivb	31	1	32	7.3
	Clivc	1		1	9.2
	Clivc_2	3		3	8.3
	Clivd	1		1	7.6
	Clivd_2	4		4	6.8
	* Nyctosyles *				
	Nycta	11	1	12	8.5
	Nyctb	21		21	9.9
	* Oxydrepanus *				
	Oxyda	1		1	2.2
	Oxydb	3		3	3.2
	Oxydc	1		1	2.6
	* Stratiotes *				
	Strasp	3		3	6.9
	** Zuphiini **	**1**	**2**	**3**	
	* Pseudaptinus *				
	Pseuapa	1		1	6.0
	Pseuapb		2	2	5.5
	**Column totals**	**564**	**691**	**1255**	

## Appendix C

**Table C1.** Carabidae tribes with higher numbers of ‘common’ morphospecies than expected. The number of morphospecies collected in this study are listed under ‘observed’, whereas the expected number of morphospecies was calculated (see methods). Analyses were conducted on the combined values for both FP and TF forests. The number of abundant morphospecies among carabid tribes were significantly different than the expected number of morphospecies (chi-square χ^2^ = 33.6, P < 0.001). Differences in the distribution of ‘rare’ morphospecies among tribes were not significantly different.

Abundant		No. of S		
*Direction of difference*	*Tribe*	* Observed *	*Expected*	*Difference*
*More than expected*	Bembidiini	3	1.99	1.01
	Cicindelini	4	0.53	3.47
	Lachnophorini	3	1.59	1.41
	Loxandrini	3	1.59	1.41
	Pentagonicini	2	0.40	1.60
				
*Approximately equal*	Scaritini	2	1.99	0.01
				
*Fewer than expected*	Lebiini	2	4.65	-2.65

## Appendix E

To determine the influence of abundance on NMDS, ordination analysis was also completed with presence/absence data using Jaccard dissimilarity values. The resulting patterns for FP and TF forest are highly similar to the results based on abundance data with the morphospecies assemblages for FP and TF forest significantly different (F = 3.86, df = 1, P < 0.001; Fig. E1). The mean Jaccard dissimilarly value between FP and TF forest was 0.87 while mean within forest type values were 0.80 for FP forest and 0.76 for TF forest. Using presence/absence data had a minimal effect on the within forest type dissimilarity values and even less so for the dissimilarity value between FP and TF forests.

## Supplementary materials

Supplementary material 1 Geolocation data for sampling sites at Tiputini Biodiversity Station Data type geospatial Explanation note Latitude and longitude in DDM format for the 24 sampling sites at Tiputini Biodiversity Station. https://binary.pensoft.net/file/555578This dataset is made available under the Open Database License (http://opendatacommons.org/licenses/odbl/1.0/). The Open Database License (ODbL) is a license agreement intended to allow users to freely share, modify, and use this Dataset while maintaining this same freedom for others, provided that the original source and author(s) are credited.Kathryn Riley Peterson
